# Influence of Human Papillomavirus E7 Oncoprotein on Maturation and Function of Plasmacytoid Dendritic Cells *In Vitro*

**DOI:** 10.1007/s12250-018-0069-3

**Published:** 2018-12-19

**Authors:** Rui Han, Yin-Jing Song, Si-Yuan Sun, Qiang Zhou, Xian-Zhen Chen, Qiao-Li Zheng, Hao Cheng

**Affiliations:** Department of Dermatology and Venereology, Sir Run Run Shaw Hospital, School of Medicine, Zhejiang University, Hangzhou, 310016 China

**Keywords:** Human papillomavirus (HPV), Plasmacytoid dendritic cells (pDCs), Toll-like receptors (TLRs), Innate immunity

## Abstract

The major difficulties of human papillomavirus (HPV) treatment are its persistence and recurrence. The HPV E7 oncoprotein-loaded dendritic cells have been evaluated as cellular vaccine in previous reports. Plasmacytoid dendritic cells (pDCs) play an essential role of connecting the innate immune response and adaptive immune response in the immune system. But they function in HPV E7 loading is unclear. To investigate whether loading of the HPV type 6b, 11, and 16 E7 proteins affects the activity of pDCs, human peripheral blood-separated pDCs and mouse bone marrow-derived pDCs were pulsed with the HPV E7 proteins. The expression levels of CD40, CD80, CD86, and MHC II were significantly upregulated in pDCs upon HPV 6b/11 E7 protein pulse. The secretion and gene expression of type I IFN and IL-6 were both upregulated by HPV 6b/11 E7 proteins, more significant than HPV 16 E7 protein. The expression of essential factors of TLR signaling pathway and JNK/p38 MAP kinase signaling pathway were all increased in HPV 6b/11 E7 proteins pulsed pDCs. Our results suggest that HPV E7 proteins could promote the differentiation and maturation of pDCs and activate the TLR and MAPK pathway to induce host innate immune response. It might be conducive to explore novel immunotherapy targeting HPV infection with HPV E7 loaded pDC.

**Electronic supplementary material:**

The online version of this article (10.1007/s12250-018-0069-3) contains supplementary material, which is available to authorized users.

## Introduction

Human papillomavirus (HPV) infection is the main cause of condyloma acuminatum, mucosa verrucous hyperplasia, cervical cancer, oropharyngeal cancer, and skin cancer, which are difficult to manage and effective control methods have been lacking until recently. Persistence and recurrence are the major difficulties associated with management of HPV infection, because effective host cellular immunity could not be induced to clear the HPV infection (Zhou *et al.*^2013^).

HPV mainly causes accretion of keratinocytes in the skin and mucosa. HPV gene or protein may interfere with the activation of host innate antiviral immunity and adaptive immunity (Einstein *et al.*^2009^). Dendritic cells (DCs) are the major cells mediating innate immunity. Plasmacytoid dendritic cells (pDCs), as an essential subset of dendritic cells, play a crucial role of connecting the innate immune response and the adaptive immune response in the immune system. pDCs could secrete type I interferon (IFN) 10–100 times more than other cell types including myeloid DC. HPV 16 VLP could induce the production of type I IFN in pDCs (Bontkes *et al.*^2005^). However, it is known that the innate immune response induced by HPV VLP infection is rather limited, which is often not sufficient to clear the virus, resulting in persistent infection. Because the E6 and E7 major oncoproteins from this DNA virus are critical in the deregulation of the cell cycle, apoptosis, and adaptive immune surveillance, they are considered to be specific targets for immunotherapy. HPV 16/18 E7 antigen-loaded DCs have been evaluated as cellular tumor vaccine in previous studies (Nonn *et al.*^2003^; Santin *et al.*^2008^). However, the HPV E7-loaded pDCs have not been studied.

During the maturation of pDC, toll-like receptors (TLRs) are indispensable in up-regulating the expression and migration of chemokines. TLRs are innate immune receptors that recognize conserved motifs on microbes and induce inflammatory signals. TLR expression of human and mouse pDC is limited to TLR7 and TLR9 (Hornung *et al.*^2002^; Edwards *et al.*^2003^). Consequently, the pDC is able to detect inactivated herpes virus (DNA virus) (Lund *et al.*^2003^; Rasmussen *et al.*^2007^) and inactivated influenza virus (ssRNA virus) (Diebold *et al.*^2004^) independent of viral replication. Therefore, it is necessary to investigate the status of TLR/MyD88 pathway in pDCs upon HPV E7 loading.

The goal of this study is to investigate the possible influence of HPV E7 proteins on the maturation and functions of pDC, and the effect on MAPK pathway. These findings may present an experimental basis for immunotherapy of HPV infection with E7-pulsed pDC.

## Materials and Methods

### Expression and Purification of HPV 16 E7 Proteins

The full-length coding sequence of the HPV 16 E7 gene was inserted into the pGEX-4T2 vector in-frame with the GST open reading frame. The HPV 16-E7 gene and pGEX-4T2 vector were cleaved by *Eco*R I (Takara) and *Bam*H I (Takara), and linked by T4 DNA ligase (Takara). The reconstructed pGEX-4T2-(HPV16E7) vector was transformed into competent *Escherichia coli* DH5α (Takara). Bacterial cells were collected from an overnight culture in lysogeny broth medium by centrifugation, washed twice with phosphate buffered saline, and lysed for plasmid isolation using the manufacturer’s protocol (OMEGA Bio-Tek). The vector was cleaved with the restricted endonucleases *Eco*R I/*Bam*H I, followed by agarose gel electrophoresis (Supplementary Figure S1) and sequencing. The constructed expression vector of pGEX-4T2-(HPV16E7) was introduced into *E coli* DH5α and induced by 0.2 mmol/L isopropyl β-D thiogalactopyranoside (IPTG; Beyotime, China), which resulted in the expression of the GST-HPV 16-E7 fusion protein. After sonication and centrifugation, the proteins were separated by sodium dodecyl sulfate–polyacrylamide gel electrophoresis (SDS-PAGE) and visualized by Coomassie blue staining (Supplementary Figure S2A). The fusion protein was further purified using glutathione-Sepharose 4B beads and the GST tag was removed with thrombin. The purity of HPV16 E7 protein was confirmed by SDS-PAGE (Supplementary Figure S2B).

The purified HPV 6b E7 and 11 E7 proteins were obtained using a method similar to that previously described in our previous publications (Tang *et al.*^2011^; Ding *et al.*^2014^).

### Human PBMC and pDCs Isolation

Peripheral blood mononuclear cells (PBMC) were isolated from HLA-A*0201 healthy donors by Lympholyte-H (Cedarlane Laboratories, Canada) density gradient centrifugation as recommended by the manufacturer. pDCs were negatively selected using Plasmacytoid Dendritic Cell Isolation Kit II (Miltenyi Biotec). Purity of sorted pDCs (85%–95%) was analyzed by staining with anti-BDCA-2 and anti-CD123 monoclonal antibodies (Miltenyi Biotec) by flow cytometry. Purified pDCs were cultured at 5 × 10^5^ cells/mL respectively with 10 ng/mL human recombinant interleukin-3 (rIL-3) in RPMI 1640 (Life Technologies) supplemented with 10% fetal calf serum (Hyclone), 2 mmol/L l-glutamine (Invitrogen), and antibiotics (penicillin–streptomycin, Life Technologies).

### *In Vitro* Differentiation of Mouse pDCs

Female C57BL/6 mice, 6–8 weeks of age, were purchased from The Animal Laboratory of Chinese Academy of Sciences (Shanghai) and housed at the Central Animal Facility of Zhejiang University. All mice studies were approved by the Institutional Animal Care and Use Committee and were performed in accordance with the institutional guidelines. Bone marrow cells were isolated by flushing femurs and tibiae of mice with RPMI 1640 medium supplemented with 10% FCS, 10 mmol/L HEPES, 1 mmol/L sodium pyruvate, 2 mmol/L GlutaMAX (Life Technologies), 100 U/mL penicillin (Life Technologies), 100 mg/mL streptomycin (Life Technologies), and 0.1 mmol/L 2-ME. Red blood cells were lysed from bone marrow cell preparations using red blood cell lysing buffer (Sigma Aldrich). Bone marrow cells were cultivated for 5 days at a density of 1 × 10^6^/mL supplemented with 100 ng/mL Flt-3L (PeproTech) for differentiation. The medium was changed once during cultures by replacing two-thirds of the medium with fresh cytokine-supplemented medium.

### Cell Viability and Proliferation Assay

Cell Counting Kit-8 (CCK-8; Dojindo Laboratories, Japan) was used to assess the rate of cellular proliferation and quantify cell viability. In brief, pDCs were seeded in 96-well plates with 100 µL of medium at a density of 2 × 10^4^ cells per well. After incubation of cells with 10 µg/mL HPV 6b/11/16 E7 protein (self-prepared as described), TLR9 ligands CpG-ODN (2216) at 2 mmol/L (Invitrogen), and TLR7 agonist imiquimod at 4 µg/mL (Invitrogen) respectively for 24 h, 10 µL of CCK8 solution was applied to each well and incubated for 1 h at 37 °C. Finally, the absorbance values at 450 nm were determined using a microplate reader (FLX800TBID, BioTek Instruments, Winooski, VT). All experiments were conducted in triplicate.

### *In Vitro* HPV Type 6b/11/16 E7 and TLRs Agonists Stimulation of pDCs

Cells were stimulated with 10 µg/mL HPV type 6b/11/16 early protein E7 along with the TLR9 ligands CpG-ODN (2216) (Invitrogen) at 2 mmol/L, or TLR7 agonist imiquimod (Invitrogen) at 4 µg/mL in 24-well plates for 24 h at 37 °C and 5% CO_2_.

### Phenotyping of pDCs Stimulated by HPV 6b/11/16 E7 Proteins and TLRs Agonists

The human pDCs and mice bone marrow cells derived pDCs were characterized using following fluorochrome-labeled human and mouse monoclonal antibodies (anti-CD40, anti-CD80, anti-CD83, anti-CD86, anti-HLA-DR, as well as their corresponding PE- or FITC-labeled isotype control antibodies, eBioscience, USA) respectively, and analyzed on an EPICSXL flow cytometer (Beckman Coulter, USA). In human pDCs, the expression of TLRs were also tested using human monoclonal anti-TLR7 and anti-TLR9 antibodies, and their corresponding PE- or FITC-labeled isotype control antibodies; eBioscience). Analysis was carried on an EPICSXL flow cytometer (Beckman Coulter).

### Cytokine Expression of pDCs by Stimulation with HPV E7 Proteins and TLR Agonists

Supernatants were collected from pDCs after 24-h stimulation with HPV type 6b/11/16 early protein E7 and TLR agonists. The expression of cytokines (IFN-α and IL-6) were determined by human and mouse enzyme-linked immunosorbent assay (ELISA) kits according to the manufacturer’s instructions (BioSource, USA). Absorption was determined with a microplate reader (SunriseTM Remote) at 450 nm.

### Transcription of the TLRs, TRAF6, IL-6 and IFN-α on TLR Pathway

Total cellular RNA was extracted from cultured pDCs with the RNA extraction kit (Qiagen). Complementary DNA synthesis was performed with 1 µg of total RNA was reverse transcribed using Oligo dT 18 (TAKARA) and Superscript II™ reverse transcriptase (Invitrogen). TaqMan Universal PCR Master Mix and predeveloped QuantiTect primer assays for human and mouse TLR7, TLR9, TRAF6, IFN-α, IL-6, and GAPDH were used in a total volume of 20 µL reaction system. Quantitative PCR was performed for 45 cycles of 95 °C for 30 s, followed by 60 °C for 1 min using Applied Biosystems 7300 (Life Technologies, Sydney, Australia). Each reaction was run in triplicate.

### Expression of the Proteins on the JNK/p38 MAP Kinase Pathways in Mouse pDCs Stimulated by HPV E7 Proteins

Total protein extracts from pDCs lysate were denatured and loaded on SDS-PAGE gels and then transferred to PVDF membranes, blocked with 5% nonfat milk in TBST buffer, and incubated with p-TBK1, TBK1, p-p65, p65, p-JNK, JNK, p38, p-p38, ERK, p-ERK (Cell Signaling Technologies), and p-IRF7, IRF7 antibody (BD Pharmingen). The band density was quantified using Image J software.

### Statistical Analyses

All samples were performed in at least duplicate, with each experiment repeated at least twice. Results are expressed as mean ± SD. GraphPad Prism was used to calculate *P* values: ***P < 0.001; **P = 0.001–0.01; *P = 0.01–0.05; and ns means *P* > 0.05.

## Results

### Cell Viability of pDCs Stimulated by HPV E7 Proteins and TLR Agonists *In Vitro*

**Fig. 1:**
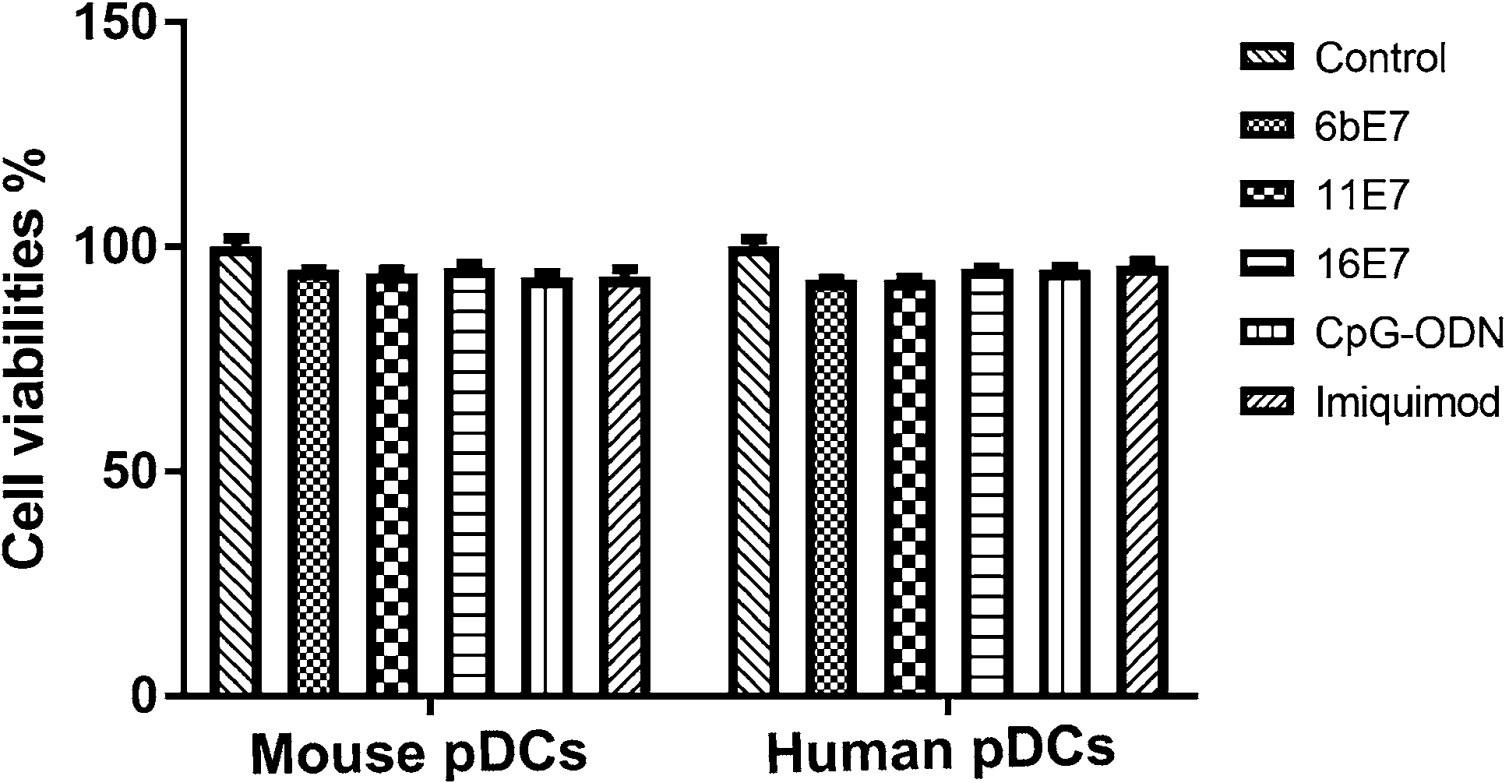
Scatter plot to show the % of viable cells relative to the untreated control cells, determined by CCK-8 (see Methods), as indicator of cytotoxicity of HPV E7 proteins and TLR agonists on mouse and human pDCs.

pDCs were incubated with HPV 6b E7, 11 E7, 16 E7 protein, CpG-ODN (2216), or imiquimod, respectively for 24 h (Fig. 1). None of them changed the viability and numbers of pDCs, excluding the cytotoxic effect of these stimuli on pDCs.

### The Maturation of pDCs Loaded with HPV E7 Protein and Costimulated with TLR Ligands

**Fig. 2:**
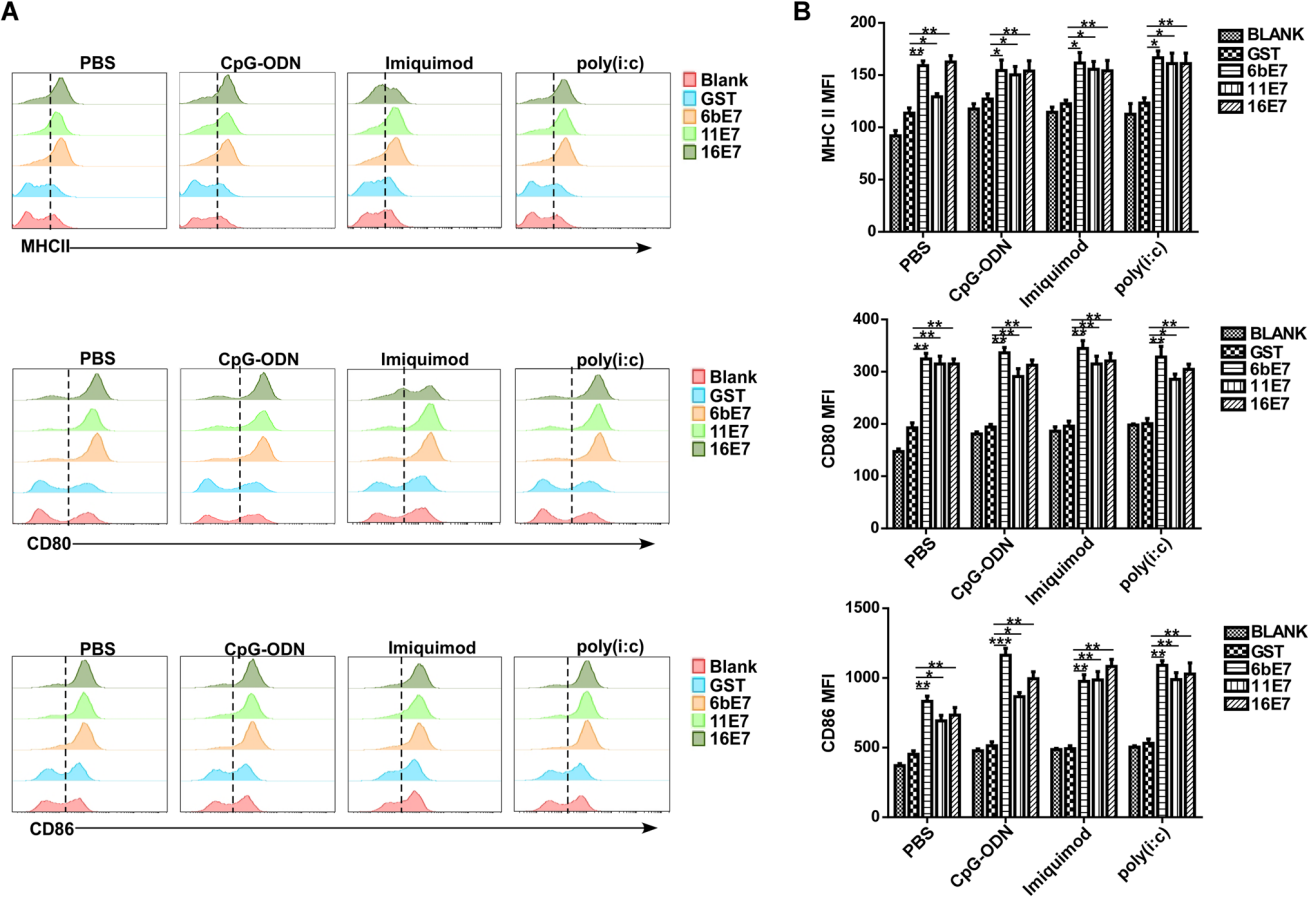
The effect of HPV E7 proteins on the expression of costimulatory molecules in mouse pDCs. Mouse pDCs were treated with indicated HPV E7 proteins for 24 h in the presence or absence of TLR agonists, and the levels of CD80, CD86, and MHC II molecules were evaluated by flow cytometry. Representative plots (**A**) and statistical results (**B**) are shown.

HPV 6b/11/16 E7 protein alone induced a significant upregulation of CD80, CD86, and MHC II expression in mouse pDCs as compared with GST controls (*P* < 0.001) (Fig. 2). Costimulation with imiquimod, CpG-ODN, or poly(I:C) caused similar effect (*P* < 0.001), but failed to further increase the expression of CD80, CD86, and MHC II compared with E7 protein stimulation alone.

**Fig. 3:**
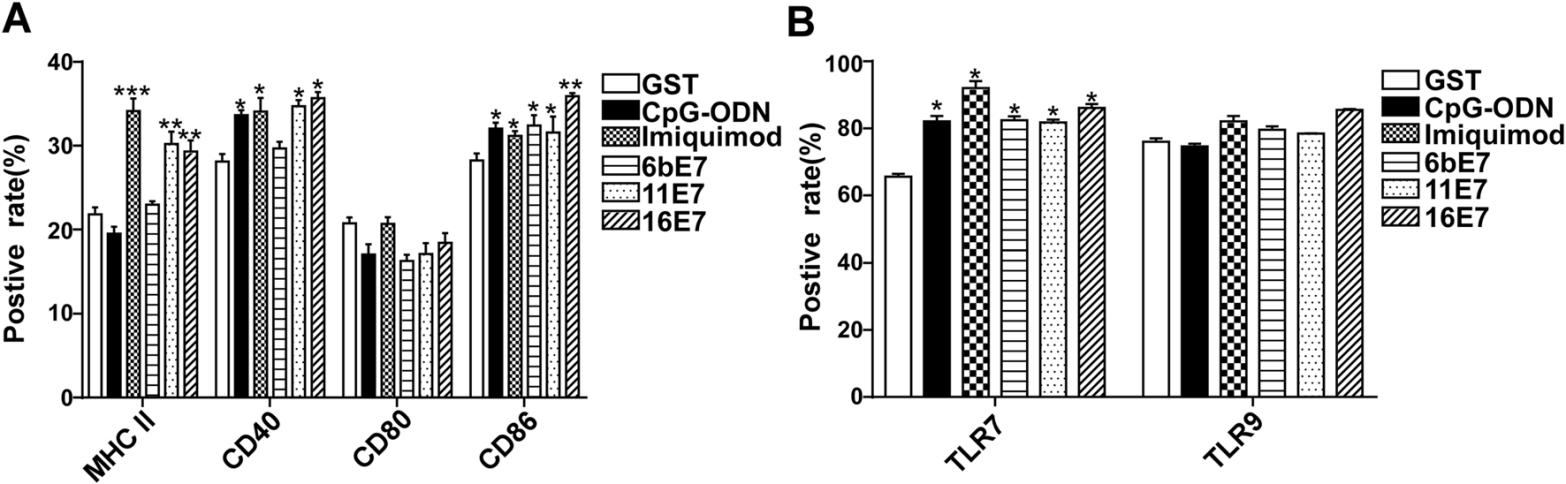
Phenotypic maturation of human pDCs and TLRs expression in human pDCs by HPV E7 proteins and TLR agonists stimulation for 24 h. **A** The expression of CD40, CD80, CD86, and MHC II of pDCs upon stimulation was detected. **B** The expression of TLR7 and TLR9 of pDCs upon stimulation was detected. Data was shown as mean ± SD.

In human pDCs, HPV 11 E7 protein alone induced a significant upregulation of CD40, CD86, and MHC II expression as compared with GST controls (Fig. 3A). In contrast, HPV 6b E7 protein can only increase CD86 expression. However, imiquimod stimulation enhanced the level of CD40, CD86, and MHC II in pDCs, but CpG-ODN stimulation can only induce an upregulation of CD40. Interestingly, we found that all of the aforementioned stimuli except for HPV 16 E7 induced a significant upregulation of TLR7, but not TLR9 expression in pDCs (Fig. 3B). No significant difference in expression of CD80, TLR9 was observed when stimulated with HPV E7 proteins alone or in combination with imiquimod or CpG-ODN.

### Cytokine Production of pDCs Stimulated by HPV E7 Proteins and TLR Agonists

**Fig. 4:**
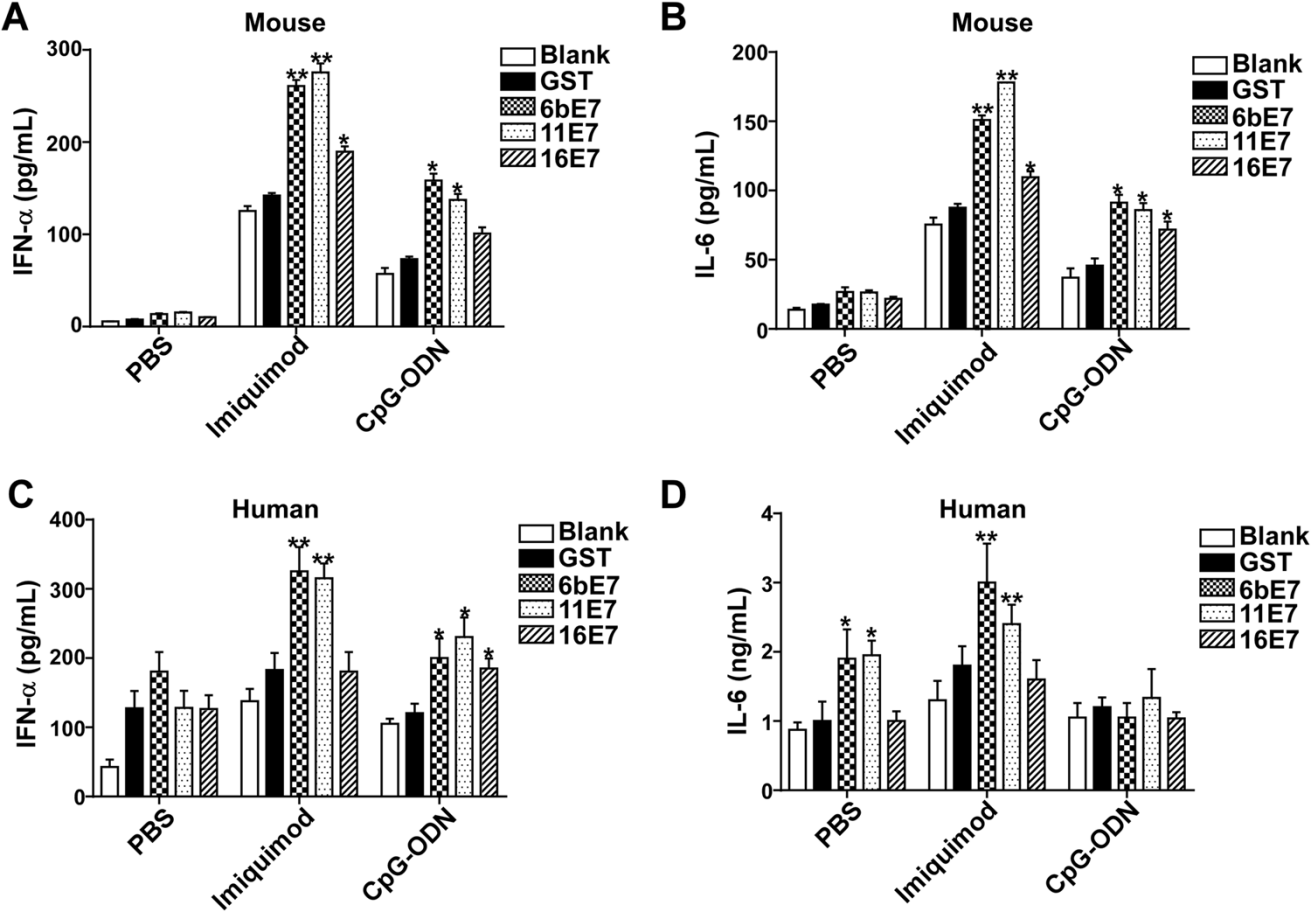
ELISA assay of IFN-α and IL-6 production in mouse (**A** and **B**) or human pDCs (**C** and **D**) stimulated by GST protein, HPV E7 proteins in PBS buffer or in combination with TLR agonists imiquimod or CpG-ODN. Data was shown mean ± SD. *P* values are indicated by * (< 0.05) and ** (< 0.01).

Activated pDC can produce various inflammatory cytokines, especially type I IFN. We found that HPV E7 proteins alone increased production of IFN-α and IL-6 in mouse pDC, which were further upregulated significantly by costimulation with imiquimod or CpG-ODN (Fig. 4A, 4B). HPV 6b and 11 E7 proteins alone could upregulate the production of IFN-α and IL-6. Costimulated with imiquimod, HPV 6b and 11 E7 proteins promoted the secretion of IFN-α and IL-6 much more significantly (*P* < 0.01). HPV 16 E7 and imiquimod costimulated overproduction of IFN-α and IL-6 (*P* < 0.05). Similarly, costimulation with CpG-ODN, HPV 6b and 11 E7 proteins upregulated the secretion of IFN-α and IL-6 significantly (*P* < 0.05). HPV 16 E7 induced the overproduction of IL-6 (*P* < 0.05) upon costimulation with CpG-ODN (*P* < 0.05), but not with IFN-α (*P* > 0.05).

This effect of HPV E7 proteins was further investigated in human pDCs (Fig. 4C, 4D). HPV E7 proteins alone could not upregulate the secretion of IFN-α. Costimulated with imiquimod, HPV 6b and 11 E7 proteins, but not 16 E7 promoted the secretion of IFN-α significantly, while upon costimulation with CpG-ODN, HPV 6b, 11 and 16 E7 proteins promoted the secretion of IFN-α significantly. Similarly, HPV 6b and 11 E7 proteins upregulated the secretion of IL-6, which was significantly costimulated by imiquimod. But, costimulated with CpG-ODN, HPV 6b, 11 and 16 E7 proteins showed no influence on the production of IL-6.

### Transcriptional Upregulation of Essential Factors of TLR Signaling Pathway by HPV E7 Proteins and TLR Agonists-Pulsed pDCs

**Fig. 5:**
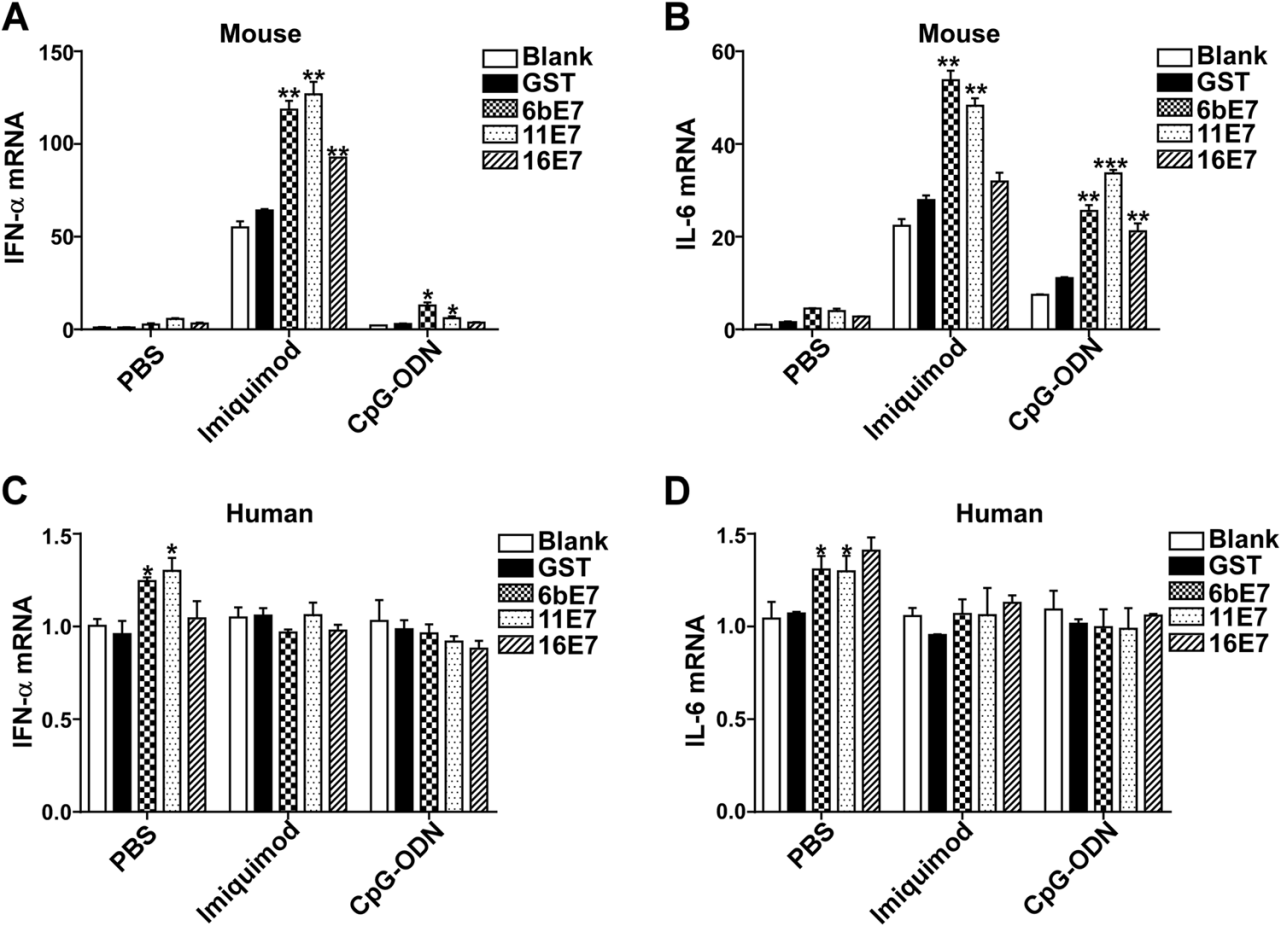
Bar charts depicting fold increase of expression of IFN-α and IL-6 mRNA in mouse (**A** and **B**) or human (**C** and **D**) pDCs, stimulated by HPV E7 proteins as single agents or in combination with TLR agonists, imiquimod or CpG-ODN, relative to blank. GAPDH was used as the internal control. Data was shown as mean ± SD. *P* values are indicated by * (< 0.05), ** (< 0.01) and *** (< 0.001).

The transcription of type I IFN and inflammatory cytokines was also examined by real-time PCR. The results of mRNA level of IFN-α and IL-6 in mouse pDCs (Fig. 5A, 5B) were consistent with the aforementioned ELISA results (Fig. 4A, 4B). The mRNA of IFN-α and IL-6 was increased in pDC stimulated with HPV E7 proteins alone, and significantly exaggerated by costimulation with imiquimod and CpG-ODN. Interestingly, the amplification effect of imiquimod is much higher than that of CpG-ODN. The costimulating effect of imiquimod and CpG-ODN on HPV 6b and 11 E7 is more significant than that of HPV 16 E7 stimulation.

In human pDCs, the overexpression of IFN-α and IL-6 was only seen by stimulation with HPV 6b and 11E7 proteins alone (Fig. 5C, 5D). However, HPV 16 E7 had no appreciable such stimulatory effect. The transcription level of IFN-α and IL-6 in human pDCs was not affected by costimulation with imiquimod or CpG-ODN.

### HPV E7 Loading Promoted the Expression of TLR7, TLR9 and TRAF6 in pDCs

**Fig. 6:**
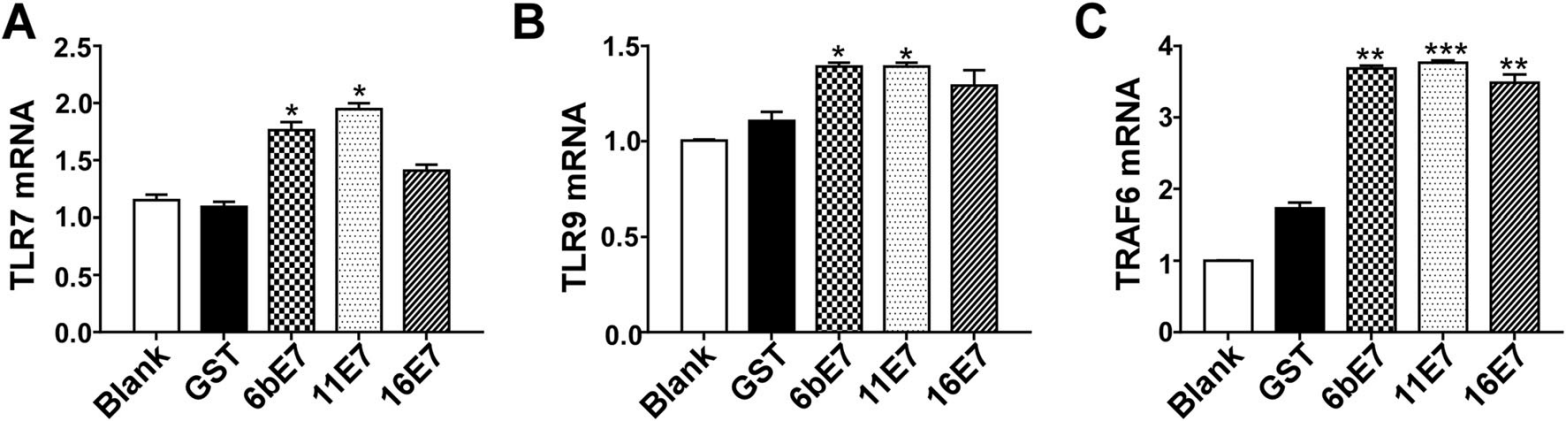
Bar charts of fold increase of expression of TLR7 (**A**), TLR9 (**B**), and TRAF6 (**C**) mRNA expression in mouse pDCs stimulated by HPV E7 proteins relative to GST protein. GAPDH was used as the internal control. Data was shown as mean ± SD. *P* values are indicated by * (< 0.05), ** (< 0.01) and *** (< 0.001) and “ns” represents *P* > 0.05.

Because TLR signaling pathway plays a crucial role on the maturation of pDCs and subsequent secretion of effector cytokines, we determined whether HPV E7 loading played a role in regulating the expression of certain components in TLR signaling. The results showed that the transcription levels of TLR7, TLR9, and TRAF6 in mouse pDCs were significantly increased following stimulation with HPV 6b and 11 E7 proteins (Fig. 6). While HPV 16 E7 had non-significant effect on the transcription of TLR7 and TLR9 (Fig. 6A, 6B), it elevated TRAF6 mRNA (Fig. 6C).

### E7 Proteins Activates the MAPK Pathways in pDCs

**Fig. 7:**
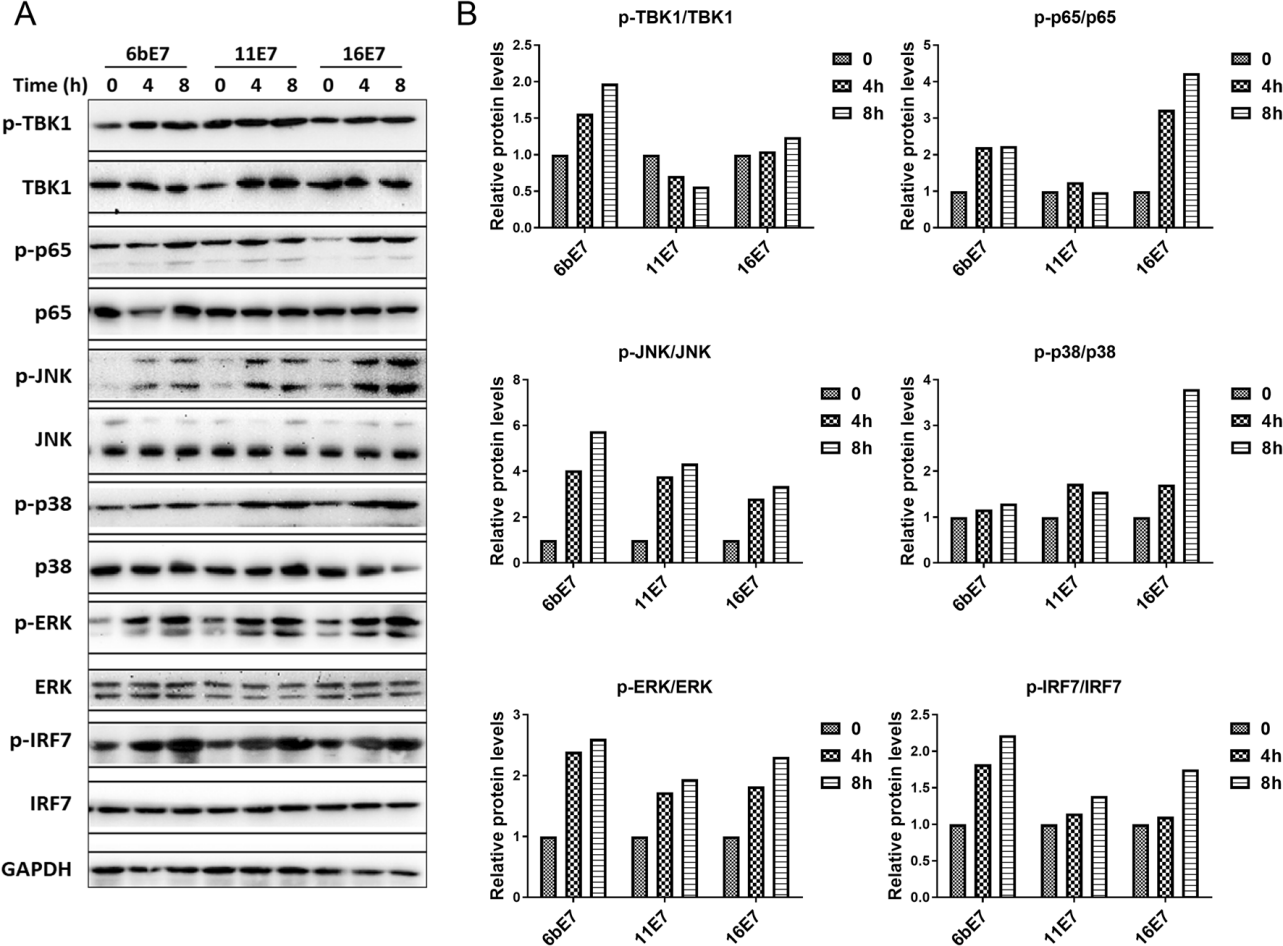
**A** Immunoblots of total and phosphorylated TBK1, NF-κB (p65), JNK, p38, ERK and IRF7 proteins in mouse pDCs following 0, 4 or 8 h of stimulation with HPV 6b E7, 11 E7 or 16 E7 in combination with imiquimod. **B** Densitometric analysis (ImageJ) of p-TBK1/TBK1, p-P65/65, p-JNK/JNK, p-P38/P38, p-ERK/ERK, and p-IRF7/IRF7.

MAPKs and NF-κB are pivotal signaling nodes downstream of TLR7/9 in pDC activation as evidenced by the effect of these cytokines on TBK1, p65, JNK, ERK, p38, and IRF7 (Scott *et al.*^1993^; Pomerantz and Baltimore 1999; Johnson and Lapadat 2002; Schmid *et al.*^2014^). For this reason, we further investigated the effects of HPV E7 proteins on the activation of these pathways. Western blot results and analysis showed that the phosphorylation of JNK, ERK, p38, and IRF7 was enhanced in pDCs treated with all the three HPV E7 proteins (Fig. 7). While HPV 6b and 16 E7 elevated the phosphorylation of TBK1 and p65, HPV-11 E7 had slightly inhibitory effect.

## Discussion

HPV early protein E6 and E7 could induce cellular immune response in DCs, leading to antiviral and antitumor immunity effects, and DC vaccine has also been explored as a clinical therapeutic vaccine (Nonn *et al.*^2003^; Santin *et al.*^2008^; Yang *et al.*^2016^). Some studies have shown that HPV E6, E7 could affect antigen presentation of DC and DC mediated T cell activation, which is indispensable in HPV immune escape (Kim *et al.*^2011^). pDCs as the potent type I IFN-producing cells are critical to eliminate viral burden from the host in antiviral immunity (Swiecki and Colonna 2015), especially at the acute phase. When viruses, such as cytomegalovirus (Del Prete *et al.*^2015^), influenza virus (Sugimura *et al.*^2015^), HIV-1 (Kaushik *et al.*^2013^), hepatitis B (Jan *et al.*^2012^), hepatitis C virus (Zhang *et al.*^2013^), herpes simplex virus-1 (Rasmussen *et al.*^2007^) and -2 (Lund *et al.*^2003^), or HPV16 (Hasan *et al.*^2007^) infect the body for the first time, pDCs can produce I type IFN to resist viral infection, and present antigen to T cells and B cells to initiate specific immune response. pDCs recognize pathogens through a battery of cell surface-localized regulatory receptors, including TLRs, C-type lectin, and Fc receptors.

In our previous study, increased pDCs were seen in the dermis of condyloma acuminatum lesion (Zhu *et al.*^2015^), but *in vitro* studies indicated no effect on pDC viabilities and proliferation pulsed by exposure to HPV E7 proteins. It may be due to the increased differentiation and maturation of pDC by HPV E7 proteins. With the HPV E7 proteins and TLR agonist stimulation, the maturation and the ability of antigen presentation function of pDCs was highly promoted, which suggested high immunogenicity of HPV E7 proteins. However, the promotion of maturation in human pDCs was not notable as in mouse pDC, perhaps because human pDCs isolated from volunteers’ blood were mixture of mature and immature pDCs, whereas, immature mouse pDCs were differentiated *in vitro* with Flt-3L prior to exposure to E7 proteins.

In addition, the secretion of type I IFN and inflammatory cytokines IL-6 was up-regulated by HPV 6b and 11 E7 proteins and further increased by the combination of imiquimod and CpG-ODN, which suggested the immune function of the pDCs was upregulated by the HPV E7 proteins. In immature pDCs, TLR7 and TLR9 reside in the endoplasmic reticulum, and the ligation with their agonists triggers a signaling cascade, which activates the assembly of a multiprotein signal-transducing complex in the cytoplasm that is composed of interleukin-1-receptor associated kinase (IRAK)1 and IRAK4, tumor necrosis factor receptor-associated factor 6 (TRAF6), and interferon-regulatory factor 7 (IRF7) (Gilliet *et al.*^2008^). The phosphorylation and nuclear translocation of IRF7 induces a large amount of type I IFN in pDCs (Honda *et al.*^2005^). Our results showed that the downstream factors TRAF6 and IRF7 could also be upregulated by HPV E7 proteins loading in mouse pDCs. Therefore, we speculate that the immune function of pDCs could be activated by HPV E7 proteins through TLR signaling pathway, which results in secretion of type I IFNs as well as production of proinflammatory cytokines and chemokines.

TLR7 senses RNA viruses, endogenous RNA, and synthetic oligoribonucleotides, whereas TLR9 detects DNA viruses containing unmethylated CpG-rich DNA sequences, endogenous DNA, and synthetic CpG oligodeoxyribonucleotides. In a previous study, we observed increased expression of TLR9 in CA lesions, which was more significant than TLR7 (Zhu *et al.*^2016^). But from the mRNA level, both TLR7 and TLR9 could be moderately upregulated by HPV 6b and 11 E7 in mouse pDCs, except HPV 16 E7 protein. This suggests low-risk HPV E7 oncoproteins may increase the TLRs transcription and expression, to enhance the TLR signaling pathways, and may induce more intensive response compared with high-risk HPV E7.

To investigate the intracellular mechanisms of how HPV E7 proteins enhance pDC responses, the levels of those molecules in p38 MAPK signaling pathway were evaluated. Interestingly, HPV E7 proteins strongly increased the phosphorylation level of both p38 and its downstream molecules JNK and ERK in mouse pDCs posttreatment in comparison with GST treatment. These observations led us to hypothesize that the p38 MAPK signaling pathway is critical for pDC maturation induced by HPV E7 proteins.

In our study, HPV 16 E7 proteins performed less efficiently in promoting the differentiation and maturation of pDCs, and the secretion of type I IFN and inflammatory cytokines IL-6, compared with HPV 6b and 11 E7 proteins, suggesting the different immunogenicity of high-risk and low-risk HPV E7.

The evolution of appropriate adaptive immune response during the course of infection, as well as vaccination, relies on the priming of innate immune cells. The conserved innate immune system senses invading pathogens and establishes the adaptive immune responses. The type I IFN response induced by pDCs is considered to be a vital part of their role in the resolution of viral infections, which has been shown to inhibit HIV-1 (Wang *et al.*^2008^). In addition to type I IFN, the induction of a cascade of cytokines followed, including TNF-α, IL-6, and IL-10, which are also associated with the generation of an adaptive immunologic response (Chang and Altfeld 2010). Our research implies, upon HPV E7 loading, pDC exhibits robust production of IFN-α, which contributes to the activation of innate immunity and elimination of the virus. Therefore, the HPV E7 vaccination might be a way of improving the host innate immune response to viral elimination.

In conclusion, HPV E7 protein loading increased differentiation, and maturation of pDCs, and their production of type I IFN and IL-6, while TLR agonists could enhance these effects. The activation of TLR and MAPK signaling pathway in pDCs may play critical roles in the process. HPV E7-loaded pDC could be explored as a potential candidate of cellular vaccines against HPV infection.

## Electronic supplementary material

Below is the link to the electronic supplementary material.

Supplementary material 1 (PDF 632 kb)
