# Supplementary material for: Influence of Human Papillomavirus E7 Oncoprotein on Maturation and Function of Plasmacytoid Dendritic Cells In Vitro
Source: Virol Sin. 2018 Dec 19;33(6):493–501. doi: 10.1007/s12250-018-0069-3 (PMC6335218; doi:10.1007/s12250-018-0069-3)
Supplement: Supplementary file 1 — Supplementary material 1 (PDF 632 kb) [file 12250_2018_69_MOESM1_ESM.pdf]

VIROLOGICA SINICA

Electronic Supplementary Material

Influence of Human Papillomavirus E7 Oncoprotein on Maturation and Function of Plasmacytoid Dendritic Cells *In Vitro*

Rui Han[1,#], Yin-Jing Song[1,#], Si-Yuan Sun[1,✉], Qiang Zhou[1], Xian-Zhen Chen[1], Qiao-Li Zheng[1], Hao Cheng[1,✉]

1Department of Dermatology and Venereology, Sir Run Run Shaw Hospital, School of Medicine, Zhejiang University, Hangzhou 310016, China

Supporting information to DOI: 10.1007/s12250-018-0069-3

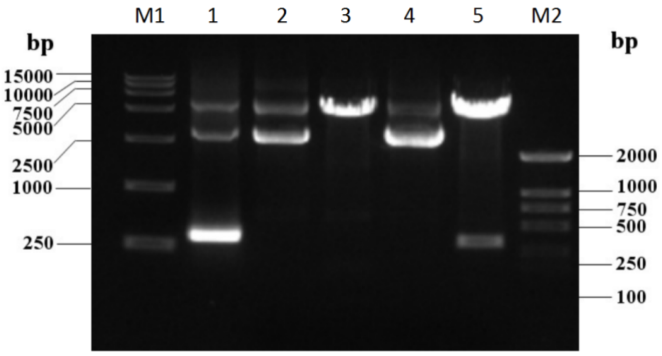

Fig. S1. Construction of the expression vector of the HPV 16 E7. The lane M1 shows the TaKaRa DL15,000 DNA marker; lane 1 shows the HPV 16 E7 gene (PCR from pGEX-4T2-(HPV16E7) vector); lane 2 is the pGEX-4T2 vector; lane 3 is the cleaved pGEX-4T2 vector by *Eco*R I and *Bam*H I; lane 4 shows the reconstructed pGEX-4T2-(HPV16E7) vector; lane 5 is the verification of pGEX-4T2-(HPV16E7) vector that was cleaved by *Eco*R I and *Bam*H I; lane M2 is TaKaRa DL 2,000 DNA marker.

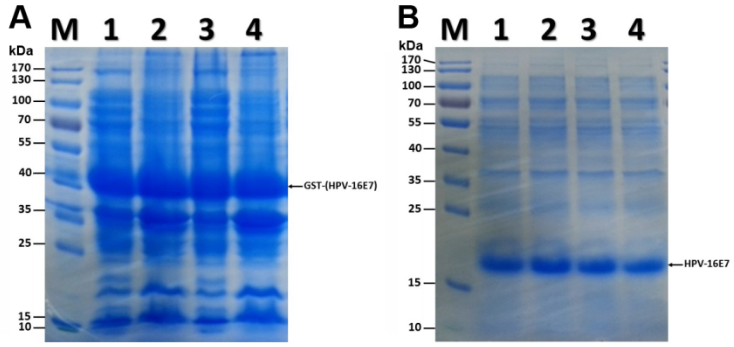

Fig. S2. Expression and purification of HPV 16 E7 protein. (A) Coomassie blue staining for HPV16 E7 fusion protein containing GST-tag. Lane M is the protein ladder. Lanes 1 and 3 show the protein of supernatant of bacteria homogenate after centrifugation. Lanes 2 and 4 show the protein of the sediment. Arrow indicates GST-HPV 16 E7 protein. (B) Coomassie blue staining for purified HPV 16 E7 proteins. Lane M is the protein ladder. Lanes 1 and 2 show the HPV 16 E7 protein without GST-tag. Lanes 3 and 4 show the purified HPV 16 E7 protein dialyzed overnight. Arrow indicates HPV 16 E7 protein.
